# Quadruple primary tumors in a lynch syndrome patient surviving more than 26 years with genetic analysis: a case report and literature review

**DOI:** 10.3389/fonc.2024.1382154

**Published:** 2024-06-04

**Authors:** Bosen Zhu, Ming Liu, Tianhao Mu, Wentao Li, Junqi Ren, Xiangtao Li, Yi Liang, Ziyi Yang, Yulin Niu, Shifu Chen, Junqiong Lin

**Affiliations:** ^1^ Department of Gastroenteroanal Surgery, The Second Affiliated Hospital of Guangdong Medical University, Zhanjiang, China; ^2^ Research and Development Division, HaploX Biotechnology, Shenzhen, China

**Keywords:** multiple primary tumors, lynch syndrome, acute non-lymphocytic leukemia, colon cancer, NGS sequencing

## Abstract

The incidence of multiple primary tumors(MPTs) is on the rise in recent years, but patients having four or more primary tumors is still rare. Lynch syndrome (LS) patients have a high risk of developing MPTs. NGS sequencing could identify the genetic alterations in different tumors to make a definite diagnosis of uncommon cases in clinical practice. Here, we report the case of a 66-year-old female patient who develops four MPTS between the ages of 41 and 66, that is sigmoid colon cancer, acute non-lymphocytic leukemia, urothelial carcinoma and ascending colon cancer. She has survived for more than 26 years since the first discovery of tumor. Targeted sequencing indicates that she has a pathogenic germline mutation in the exon 13 of *MSH2*, and her 2020 ureteral cancer sample and 2023 colon cancer sample have completely different mutation profiles. To the best of our knowledge, this is the first case of multiple primary tumors with an acute non-lymphocytic leukemia in LS patients.

## 1 Introduction

With the improvement of living conditions and medical standards in modern times, the incidence of MPTs is on the rise in recent years (1). Even so, patients having four or more primary tumors is still rare, and accounts for only 0.05% (2). MPTs are defined as two or more different primary tumors in the same individual, and they could occur synchronously or metachronously (3). Risk factors for MPTs include inherited predispositions, lifestyles, environmental factors and so on (4).

LS is an autosomal dominant disorder characterized by germline mutations in the DNA mismatch repair (MMR) system genes, such as *MLH1*, *MSH2*, *MSH6*, and PMS2 (5). The majority of LS-associated cancers exhibit deficient MMR (dMMR) and high-level microsatellite instability (MSI-H), which are key molecular features. LS is the most common hereditary cause of colorectal cancer (CRC), accounting for approximately 3% of newly diagnosed cases (6). Additionally, LS significantly increases the risk of developing multiple primary tumors.

In this report, we present a rare case of quadruple primary tumors in a LS patient with a metachronous hematologic malignancy and three solid tumors, highlighting the potential association between LS and the occurrence of hematologic malignancies.

## 2 Case description

**Figure 1:**
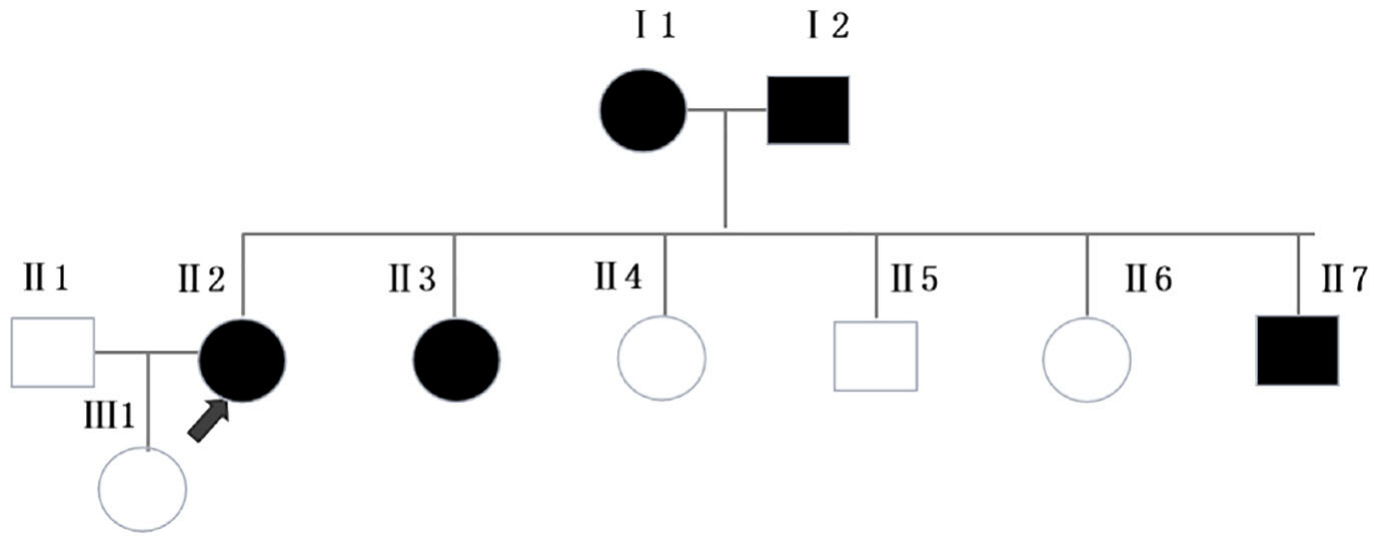
Pedigree of the family. Squares and circles represent men and women, respectively. Solid symbols represent cancer patients. Roman numerals indicate generations. Arrows point to the cancer patient.**Ⅰ1** had colon cancer at the age of 60. **Ⅰ2** had liver cancer at the age of 71. **Ⅱ3** and **Ⅱ7** had brain tumors at the age of 57 and 39, respectively.

This patient is an 66-year-old female from Zhanjiang, Guangdong province, and she is still alive now. She developed four MPTS between the ages of 41 and 66, and endured four surgeries, three for primary tumors, and one for uterine fibroids (**Table 1**). She had been working as a chef for many years, had no bad habits such as smoking or alcohol drinking, and kept a healthy diet. She had a history of hypertension for 1 year and diabetes for 3 years, and was treated with oral dapagliflozin, gliclazide, and amlodipine. The patient’s father had liver cancer at the age of 71, and her mother had colon cancer at the age of 60. She had five siblings, among whom one young brother and one young sister had brain tumors at the age of 39 and 57 years old, respectively. The patient also has a daughter who is currently in good health (**Figure 1**).

**Table 1:** The medical history of the patient.

Year	Age	Disease	Pathological diagnosis	Localization	Treatment	NGS
1997	41	Colon cancer	mucinous adenocarcinoma	Sigmoid colon	Radical surgery	No data
1998	42	Uterine fibroids	benign	Uterus	Myomectomy	No data
2007	55	Acute non-lymphocytic leukemia	M6	Blood	Chemotherapy : MA+DA+HA	No data
2020	63	Urothelial carcinoma	papillary carcinoma	Left ureter	Radical surgery	yes
2023	66	Colon cancer	Tubular adenocarcinoma	Ascending colon	Radical surgery	yes

**Figure 2:**
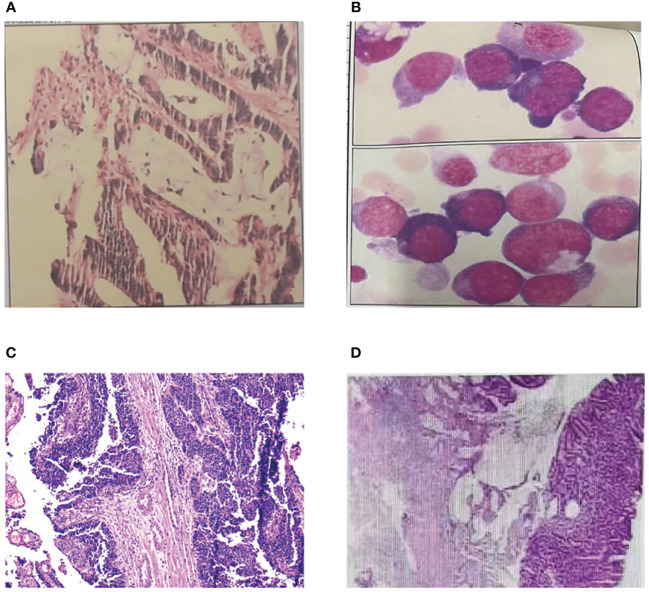
Pathological examination of MPTs. **(A)** Pathological finding of sigmoid colon cancer. **(B)** Pathological finding of acute non-lymphocytic leukemia. **(C)** Pathological finding of urothelial carcinoma. **(D)** Pathological finding of ascending colon cancer.

In the past thirty years, the patient had a history of multiple primary cancers. In 1997, the patient was admitted to Central People’s Hospital of Zhanjiang due to mucinous bloody stools for more than 4 months. Physical examination showed an 3cm×3cm hard mass with uneven surface and cross degree of motion could be felt, and it was mild and tender in her left lower abdomen. After completing all relevant examinations and making preparation for surgery, a partial colectomy of the sigmoid colon was performed. Postoperative pathological examination showed mucinous adenocarcinoma of the sigmoid colon infiltrating the outer muscular layer, and no cancer was found on both ends of the sigmoid colon (**Figure 2A**). Postoperative adjuvant chemotherapy was subsequently given. The patient was generally in good condition after these treatments. In 1998, the patient underwent surgery for uterine fibroids.

In 2007, the patient was admitted to our hospital due to pale complexion, fatigue, and shortness of breath after activity. Bone marrow aspiration showed significant hyperactivity of bone marrow, with myeloid series accounting for 30% and erythroid series accounting for 54%. The myeloid-to-erythroid ratio was 0.56:1. Abnormal proliferation of the myeloid series, mainly composed of immature granulocytes (types I+II), accounted for 19.5%, non-erythroid cells (NEC) classification accounts for 42.4%, with varying cell sizes and a few (types I+II) granules, occasionally accompanied by Auer rods. Abnormal proliferation of the erythroid series was observed, with various stages of megaloblastic changes in red blood cells and binucleated red blood cells, as well as nuclear division, nuclear lobation, and punctate basophilia. Out of 100 WBCs, 7 are nucleated red blood cells. The final diagnosis was acute non-lymphocytic leukemia, M6 type (**Figure 2B**). The patient received one cycle of MA chemotherapy, and bone marrow examination showed complete remission. Subsequently, the patient underwent four cycles of consolidation therapy with MA, DA, HA regimens, and the treatment has shown favorable efficacy.

In 2020, the patient was admitted to our hospital with complaints of abdominal pain with no obvious cause. Physical examination showed her left upper abdomen was palpated with a lump about 10cm in diameter, the liver, spleen and subcostal were not reached, the mobile dullness was negative, and the bowel sounds were normal. Subsequently, she was diagnosed with ureteral cancer and underwent laparoscopic radical surgery for left ureteral cancer. The pathological examination revealed invasive papillary carcinoma of grade II to III in the deep muscle layer of the left ureter with involvement of blood vessels (**Figure 2C**). There was hemorrhagic necrosis in the renal pelvis, but no infiltration of cancer tissue was observed in the kidney. No lymph node metastasis was found around the kidney. The postoperative outcome was favorable.

On February 28, 2023, the patient visited our hospital with a chief complaint of experiencing rectal bleeding for 5 days. The blood was mixed with the stools and appeared dark red in color. After the patient’s admission, we conducted a series of examinations. Based on the relevant test results, the patient’s medical history, and family history, we highly suspected the presence of colorectal cancer. We performed a right hemicolectomy and periintestinal lymph node dissection, which is a radical curative surgery for colon cancer patient. The pathological findings revealed tubular adenoma with high to moderate differentiation (adenocarcinoma) (**Figure 2D**), with cancer tissue infiltrating through the mucosal muscle layer into the submucosa, with a measured depth of infiltration of approximately 2.3mm. The tumor budding grade was classified as high-grade (>10 buds/0.785mm^2^). Immunodeficient analysis showed a normal expression of MLH1 and PMS2 proteins, a very reduced expression of MSH6 and the absence of MSH2 protein. The nuclear expression of MSH2 and MSH6 proteins in cancer cells confirms the possibility of mismatch repair deficiency (MMR-D) in the tumor DNA, suggesting MSI-H and MSH2 germline mutation (Lynch syndrome).

To further confirm the germline *MSH2* status and study the genomic profiling of tumors, we performed targeted sequencing on her peripheral blood leucocytes, paraffin sections of her 2020 ureteral cancer sample and the 2023 colon cancer sample using the HapOnco StarPanel NGS Assay (HaploX Biotechnology, Shenzhen). Using the Illumina NovaSeq 6000 sequencing platform, we conducted high-throughput sequencing on 680 tumor-related genes. The detected mutation forms include single nucleotide variants (SNVs), small insertions and deletions (INDELs), copy number variations (CNVs), and rearrangements (fusions). In the germline, we identified a heterozygote pathogenic variant, p.R737Nfs*18, in *MSH2* gene. It occurred in exon 13 and was not within the known functional domain. This mutation caused a frameshift of the encoded amino acid at position 737, which might affect the function of the protein. For the somatic mutations detected in these two tumors, NGS results demonstrated a different mutation landscape. we identified 77 and 116 SNV/INDELs in the 2020 ureteral cancer sample and the 2023 colon cancer sample (**Supplementary Table 1**), respectively. Mutations with clear or potential clinical significance of them were displayed in **Table 2**. These two tumor samples shared only one mutation, that is *ACVR2A* p.K437Rfs*5. The tumor mutational burden (TMB) of the 2020 ureteral cancer sample was 48.23 Muts/Mb, which ranked 1.21% in patients with the same cancer type. The TMB of the 2023 colon cancer sample was 63.12 Muts/Mb, which ranked 0.77% in patients with the same cancer type. Moreover, The microsatellite instability (MSI) status of these two tumors were all classified as highly unstable. We further selected mutated genes for KEGG enrichment analysis. The mutant genes of the 2020 ureteral cancer sample were mainly enriched in the JAK−STAT and Notch signaling pathways, while the 2023 colon cancer sample mainly in the PI3K−Akt, Ras and MAPK signaling pathways.

**Table 2:** Somatic mutations with clear or potential clinical significance of the 2020 ureteral cancer sample and the 2023 colon cancer sample.

Sample	Gene	Transcript	Exon	Nucleotide change	Alteration	Mutant allele frequency (MAF)
Urothelial carcinoma	FGFR3	NM_000142.4	7	c.742C>T	p.R248C	46.69%
	ERCC5	NM_000123.3	8	c.1616A>T	p.H539L	25.50%
	ATR	NM_001184.3	17	c.3402del	p.F1134Lfs6	24.32%
	MSH2	NM_000251.2	14	c.2254A>T	p.R752	21.25%
	RAD50	NM_005732.3	13	c.2165del	p.K722Rfs14	19.39%
	RAD54L	NM_003579.3	4	c.223C>T	p.R75	15.28%
Ascending Colon cancer	PTEN	NM_000314.6	6	c.517C>T	p.R173C	8.50%
	APC	NM_000038.5	7	c.694C>T	p.R232	8.25%
	TP53	NM_000546.5	8	c.844C>T	p.R282W	8.17%
	APC	NM_000038.5	16	c.3473_3474del	p.R1158Tfs5	7.58%
	PTEN	NM_000314.6	8	c.968del	p.N323Mfs21	3.10%
	PTEN	NM_000314.6	5	c.389G>A	p.R130Q	2.09%

mutant allele frequency (MAF): The proportion of variant reads for a given mutation. MAF is often substituted by variant allele frequency (VAF), which carries the same meaning. For tumor tissue DNA, threshold for sequencing analysis were set as follows: The variant allele frequency (VAF) threshold was set to 2%, somatic variants (SNVs or indels) presented at least 5 unique reads, at least 1 on each strand, and less than 0.5% mutant allelic frequency in the paired normal sample were retained.

## 3 Discussion

In this case report, we described a metachronous occurrence of four primary tumors. From pedigree analysis, we found the patient had a familial predisposition. Further immunohistochemical staining of her 2023 colon cancer sample showed loss of expression of MSH2 protein and a very reduced expression of MSH6 protein, implying a germline mutation within *MSH2*, as MSH6 was unstable in the absence of MSH2. The following genetic testing confirmed she had a germline frameshift mutation in the exon 13 of *MSH2* actually. The mutation falled outside the known functional domain, causing a frameshift starting at the 737th amino acid encoded, which probably affected protein function. Although this variant had not been recorded in the Clinvar database yet, a downstream truncation variant p.Val821fs had been recorded as pathogenic. Its frequency in the population genome database was extremely low. Based on these analyses, this mutation was suspected to be pathogenic. Therefore, she was clearly diagnosed with LS. The NGS results of the 2020 ureteral cancer sample and the 2023 colon cancer sample showed their mutation profiles were almost completely different, supporting MPTs. The last tumor occurred just within three years of the previous one, suggesting that patients with LS should be closely followed up in clinical practice.

The NGS method was not only a definitive way for LS diagnosis, but also provided useful information in informing personalized treatment strategies. For her 2020 ureteral cancer sample, we identified one somatic mutation with clear clinical significance, that was *FGFR3* p.R248C, which conferred a gain of function to the FGFR3 protein and led to increased activation of the MAPK signaling pathway. The Food and Drug Administration (FDA) approved erdafitinib (Balversa, Janssen Biotech) for locally advanced or metastatic urothelial carcinoma with this mutation. Besides, we identified five somatic mutations with potential clinical significance concurrently. They were *ERCC5* p.H539L, *ATR* p.F1134Lfs*6, *MSH2* p.R752*, *RAD50* p.K722Rfs*14 and *RAD54L* p.R75*. Interestingly, all of these genes were involved in the DNA Damage Response (DDR) signaling pathways, and genes harboring these mutations predicted to lead to loss of function. A prospective trial studying the relationship between *DDR* mutations and PD-1/PD-L1 blockade found that in patients with metastatic urothelial carcinoma treated with atezolizumab or nivolumab, those carrying known potentially pathogenic *DDR* mutations had an objective response rate (ORR) of 80% (7). What’s more noteworthy was that this tumor sample was MSI-H and TMB-H. Pembrolizumab had received full FDA approval for use in adults and pediatric patients with unresectable or metastatic high microsatellite instability (MSI-H) or mismatch repair deficient (dMMR) solid tumors. Similarly, the FDA had approved Pembrolizumab for the treatment of adults and pediatric patients with unresectable or metastatic tumor tissue sample with high TMB (TMB-H≥10 mutations/Mb), who have progressed following prior treatment and who had no satisfactory alternative treatment options (8). For her 2023 colon cancer sample, consistent with her 2020 ureteral cancer sample results, it was also MSI-H and TMB-H. In addition, we identified six mutations with potential clinical significance. They were *PTEN* p.R173C, *PTEN* p.N323Mfs*21, *PTEN* p.R130Q, *APC* p.R232*, *APC* p.R1158Tfs*5 and *TP53* p.R282W. Three *PTEN* gene mutations probably led to loss of function. In a preclinical study, Capivasertib (AZD5363) was shown to inhibit the growth of various solid tumor cells with *PTEN* inactivation mutations (9). Two *APC* gene mutation also predicted to bring about loss of function. In a preclinical study, vandetanib reduced the number of tumors induced by dextran sulfate sodium in an APC-deficient colon cancer mouse model (10). *TP53* p.R282W could reduce the activation of TP53 target genes and inhibit the AMPK signaling pathway. In a Phase I clinical trial, Among patients with P53 mutations receiving AZD1775 treatment., the effective rate of having a stable disease for more than 6 weeks or achieving partial remission was 21%, while among patients with wild-type P53, the effective rate was only 12% (11).

For the screening or diagnosis of LS, it is essential to recognize the limitations of traditional methods. Although Amsterdam and Bethesda criteria are two widely accepted and recognized consensus guidelines, many patients with a germline mutation in a MMR gene do not meet these criteria. Especially under China’s previous family planning policy, the drastic decrease in family members made it difficult to meet the requirement. While with the reduction of sequencing costs and the improvement of sequencing technology, more and more patients with suspected hereditary CRC now undergo LS screening or diagnosis through genetic testing. Moreover, it is important to note that the spectrum of cancers associated with LS continues to expand with the increasing use of next-generation sequencing technology. Schwark et al. conducted an analysis of germline and somatic data from over 15,000 tumors (12), covering more than 50 cancer types. They discovered that half of all MSI-H/MMR-D cancers in patients with germline *MMR* gene variants were non-colorectal and non-endometrial primary cancers. These findings highlight the necessity of evaluating the germline status of all MSI-H/MMR-D tumors, even in cancer types that are not typically associated with LS, unless biallelic somatic *MMR* gene inactivation has been identified.

MPTs refer to the occurrence of two or more primary tumors simultaneously or at different times in one or more tissues or organs within the same individual. Many multiple primary tumors have distinct genetic factors, with different gene mutations and varying cancer sites (13–19). Due to mutations in the MMR genes, patients with LS have a higher likelihood of developing various types of cancers compared to the general population (20). In addition to the classic association with CRC, LS is also associated with a variety of extraintestinal tumors. Many studies have reported extraintestinal tumors associated with Lynch syndrome, including endometrial cancer, ovarian cancer, gastric cancer, pancreatic cancer, urothelial carcinoma, bladder cancer, and prostate cancer (21–27). Different types of gene mutations may predispose LS to primary tumors in different locations (28). Mutations in the *MSH2* gene are specifically linked to an increased risk of extracolonic cancers, particularly endometrial cancer (29).

Establishing the diagnosis of an additional primary tumor can be challenging in patients with a history of previous cancer and potentially prior anticancer therapy. For instance, newly developed metastases could arise from the initial cancer diagnosis or be indicative of a second malignancy. Recognizing these situations and conducting appropriate investigations is crucial in daily clinical practice due to their significant implications for subsequent therapeutic management strategies. The application of NGS has the potential to bring about a revolutionary transformation in the diagnosis of Lynch syndrome. This can be achieved through the facilitation of NGS-based evaluation of tumor specimens for MSI screening, as well as the increasing accessibility of NGS-based multi-gene panels for direct germline testing (30).

Reports indicating occurrences of leukemia in LS patients are infrequent. However, Constitutional Mismatch Repair Deficiency Syndrome (CMMRD) is commonly associated with leukemia in individuals with MMR defects (31). CMMRD refers to patients and/or families with biallelic mutations of the DNA MMR genes. These patients typically exhibit café au lait spots, early onset of colorectal neoplasia or other LS-related cancers during childhood or adolescence, and oligopolyposis in the small bowel and/or colon, brain tumors, and hematologic malignancies (32). CMMRD is associated with homologous germline mutations, while LS is associated with heterozygote germline mutations. Additionally, Self.C et al. reported seven cases of LS in teenagers (33), all of whom were confirmed to have a monoallelic germline variant in an MMR gene (without evidence of CMMRD). Among these cases, one had acute lymphoblastic leukemia (ALL) with genotypes involving *MSH2* deletion in exon 1-6. However, it is regrettable that routine sequencing for mutational burden is not performed, so the contribution of germline MMR variants in their cohort remains unclear. Further research is needed to investigate the relationship between MMR defects in LS and leukemia, both in adults and teenagers. Our report is the first case of MPTs with an acute non-lymphocytic leukemia in LS patients.

Unfortunately, we no longer have access to the 1997 colon cancer and the 2007 acute non-lymphocytic leukemia samples because too much time has passed. Therefore, we couldn’t do any deeper for all her tumor samples, especially in exploring the association of acute non-lymphocytic leukemia with LS.

## 4 Conclusion

In conclusion, this is the first report of MPTs with an acute non-lymphocytic leukemia in LS patients. Acknowledging the limitations of traditional methodologies, the NGS approach in LS diagnosis is garnering wide recognition among clinicians. This method not only broadens the understanding of LS occurrence in various cancer types beyond the typical LS-related ones, but also provides invaluable insights for crafting personalized treatment strategies. Meanwhile, it is also the basis for the diagnosis of MPTs. For patients diagnosed with LS, consistent and close surveillance is absolutely crucial.

## Data availability statement

The raw sequence data reported in this paper have been deposited in the Genome Sequence Archive (Genomics, Proteomics & Bioinformatics 2021) in National Genomics Data Center (Nucleic Acids Res 2022), China National Center for Bioinformation / Beijing Institute of Genomics, Chinese Academy of Sciences (GSA-Human: HRA006639) that are publicly accessible at https://ngdc.cncb.ac.cn/gsa-human.

## Ethics statement

The studies involving humans were approved by The Second Affiliated Hospital of Guangdong Medical University. The studies were conducted in accordance with the local legislation and institutional requirements. The participant provided her written informed consent to participate in this study. Written informed consent was obtained from the individual for the publication of any potentially identifiable images or data included in this article.

## Author contributions

BZ: Writing – review & editing, Writing – original draft, Conceptualization. ML: Writing – review & editing, Writing – original draft, Conceptualization. TM: Writing – review & editing, Supervision, Project administration. WL: Writing – review & editing, Supervision, Methodology. JR: Writing – original draft, Supervision, Methodology, Data curation. XL: Writing – original draft, Investigation, Data curation. YL: Writing – original draft, Supervision, Methodology, Data curation. ZY: Writing – original draft, Validation, Supervision, Project administration, Methodology, Data curation. YN: Writing – original draft, Investigation, Formal analysis. SC: Writing – review & editing, Supervision, Resources, Project administration. JL: Writing – review & editing, Writing – original draft, Supervision, Resources, Methodology, Investigation, Funding acquisition, Conceptualization.
